# Engaging with Raman Spectroscopy to Investigate Antibody Aggregation

**DOI:** 10.3390/antib7030024

**Published:** 2018-07-07

**Authors:** Ilokugbe Ettah, Lorna Ashton

**Affiliations:** Department of Chemistry, Lancaster University, Lancaster, Lancashire LA1 4YB, UK; i.ettah@lancaster.ac.uk

**Keywords:** Raman spectroscopy, antibody, aggregation, protein structure

## Abstract

In the last decade, a number of studies have successfully demonstrated Raman spectroscopy as an emerging analytical technique for monitoring antibody aggregation, especially in the context of drug development and formulation. Raman spectroscopy is a robust method for investigating protein conformational changes, even in highly concentrated antibody solutions. It is non-destructive, reproducible and can probe samples in an aqueous environment. In this review, we focus on the application and challenges associated with using Raman spectroscopy as a tool to study antibody aggregates.

## 1. Introduction

The past 30 years have seen increasingly rapid growth in the number of approved antibody-based biopharmaceutical products. Over 60 products have been approved for therapeutic use and approximately 50 more are currently in late stage clinical development [1,2,3,4]. Despite this success, antibody production is still laden with challenges that impact the time and cost of development [5,6,7,8]. One key issue is the control and understanding of aggregation, which can compromise not only product quality but also safety; in particular, the propensity of aggregates to trigger immune responses [7,8,9,10]. Antibody aggregates can occur in various forms and there is currently no standard definition for an aggregate [11]. Those with altered higher order structures (non-native aggregates) are often defined as a net irreversible cluster of protein monomers that have lost some or all of their native folded structure [12,13,14,15,16]. One key advantage of Raman spectroscopy is that spectral variations including peak intensities, peak position or shape, can be directly related to changes in protein secondary and tertiary structure. This unique sensitivity to structural changes in unfolded or aggregated proteins makes Raman Spectroscopy a powerful technique for the prediction and monitoring of antibody aggregation at all stages of drug production [6,17,18,19,20,21].

Raman spectroscopy is non-invasive, non-destructive and can be used to study samples in solid and liquid forms. There is also flexibility with the choice of laser excitation wavelength, making it possible to obtain selective information from the samples. More importantly, there is no specific requirement to dilute samples to lower concentrations; therefore, Raman spectroscopy is suitable for high or low sample concentrations. Unlike more frequently utilized spectroscopic techniques such as circular dichroism, ultra-violet absorption and fluorescence where high concentrations can saturate spectral signals, in Raman spectroscopy, high concentrations give stronger signals and can actually improve the quality of the spectra [22,23]. High-quality spectral data offers the opportunity to monitor structural perturbations in test conditions that are relevant to practical usage rather than extrapolating from dilute solutions that may have a different behavior [13,24]. The current understanding of the degree to which protein structural changes influence and define aggregation is limited [12,25,26,27]. Developing and employing analytical approaches, such as Raman spectroscopy, which overcome existing limitations, is an effective way to bridge this gap and expand the knowledge of aggregation mechanisms from a structural perspective [6,8,11,28].

## 2. Raman Theory

**Figure 1:**
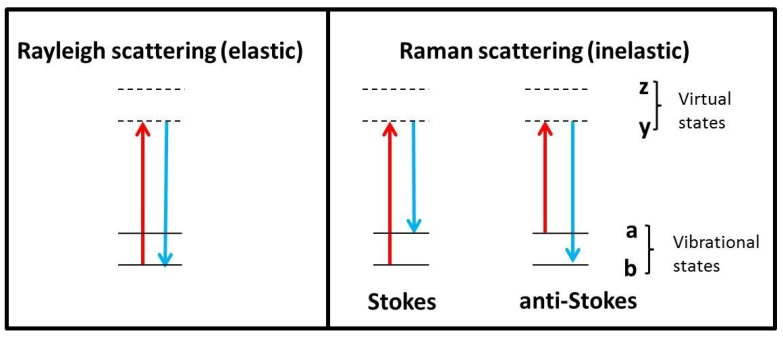
Schematic showing Rayleigh and Raman scattering processes (Stokes and anti-Stokes).

Raman spectroscopy is the inelastic scattering of light from molecules in the presence of a monochromatic light source [29,30]. When light (or photons) interact with a molecule, it can induce a transition in energy state that leaves the molecule in an excited vibrational state. The majority of scattered photons from the molecule are scattered without any change in energy (elastic or Rayleigh scattering). A small fraction of photons however, will be scattered at a different energy from that of the incident light. This effect is referred to as inelastic or Raman scattering. This difference in scattered frequencies, or Raman shift, is measured in units of cm^−1^ (wavenumber which is equal to 1/wavelength of the Raman shift). It is a relatively weak process, with a probability of 1 in 10^6^−10^8^ photons being scattered this way [29,30]. The frequency of the Raman scattered photon can be either less than the incident frequency (Stokes scattering) or greater than the incident frequency (anti-Stokes scattering) (Figure 1). Before spectra collection, the instrument is usually calibrated using materials that have strong Raman peaks such as silicon (520 cm^−1^) and diamond (1332 cm^−1^) [30]. These materials are either integrated into the instrument or may be external. Ideally, the choice of material should have Raman peaks in the same wavenumber range of the sample to be examined. The process of collection is optimized using spectral parameters such as laser power, exposure time, and number of accumulations [30,31].

**Figure 2:**
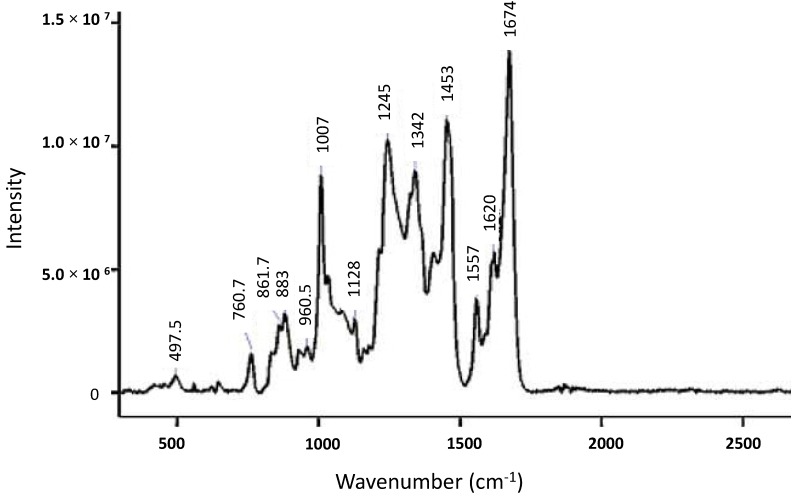
Antibody Visible Raman spectrum of a monoclonal antibody (2 mg/mL) in aqueous solution at neutral pH excited with 532 nm laser line. Reproduced with permission from [17]. Copyright Elsevier (2007).

The Raman shift results in a unique spectrum for each molecule according to the chemical bonds within it, with peaks corresponding to the vibrational frequencies of different functional groups. In the case of proteins, including antibodies, the Raman spectrum contains large amounts of structural information as there are 3*N*-6 vibrational modes, where *N* is the number of atoms, for a nonlinear molecule [30]. A typical Raman spectrum of a protein can be divided into regions identifying backbone structure, side chain environments as well as specific secondary structural elements. These regions can be further divided into the amide I region (~1630–1700 cm^−1^) assigned to C=O stretching of carbonyl groups, the amide III region (~1230–1340 cm^−1^) assigned to NH bending and C_α_–N stretching modes, while the backbone skeletal stretch (~870–1150 cm^−1^) is assigned to C_α_–C, C_α_–C_β_, C_α_–N stretches [32,33,34]. An example of a Raman spectrum of a monoclonal antibody is shown in Figure 2, demonstrating the wide range of structural information available from Raman spectra of proteins with the potential to provide unique insights into the aggregation process. 

However, two well-known limitations of the Raman effect are its intrinsic weakness and the interference of fluorescence. The drive to overcome these challenges has largely influenced significant technological advances that has transformed modern day Raman instrumentation. Essentially there has been an increase in the capability, efficiency and variety of lasers, low noise detectors, high quality optical systems including confocal microscopes that seek to maximize the scattering process [35,36,37]. As a consequence of advances in instrumentation, there are a number of variant Raman techniques, which apply the same basic principles as shown in Figure 1, but have instrument settings or adaptations that are designed to enhance the signal in a specific way or provide specific information [20,30,31]. Two such techniques that have been distinctly applied to aggregation studies include Raman optical activity (ROA) and deep UV-resonance Raman spectroscopy (DUVRR).These techniques are discussed in greater detail in later sections. Key advantages of DUVRR include low fluorescence and enhanced signals [20,30]. However, photodegradation may occur, but is usually reduced through measures that limit constant exposure of the sample to the laser beam such as spinning the sample during spectra collection as well as by reducing exposure time [30]. Although ROA has an extremely weak Raman signal and requires farlonger acquisition times than conventional Raman techniques, it uniquely provides structural information about the conformation of chiral molecules, including proteins [38,39].

Regardless of the Raman technique applied in the study of protein aggregates, a large amount of information can be contained within the spectral data set. In addition, heterogeneous samples like formulations and proteins in the presence of buffers could swamp Raman signals or create a complex spectrum with overlapping peaks, making it difficult to interpret. Therefore, chemometric methods are often required to extract and analyze the volume of information that may exist in the acquired spectra. In antibody aggregation studies, common methods include difference spectra, principal component analysis (PCA), partial least squares (PLS) and multivariate curve resolution (MCR) [22,23,40]. The application of chemometrics to Raman data is a broad area of research. It generally includes aspects such as spectral variance analysis, data pretreatment, sample variance analysis, classification, spectral deconvolution and quantitative regression analysis [20,41]. Several other methods are available for these processes, including pre-processing methods (baseline correction, cosmic ray removal, smoothing, de-noising and normalization); feature extraction methods such as linear discriminant analysis (LDA) and classification methods including linear discriminant classification (LDC), and hierarchical clustering analysis (HCA) [31,42]. For more extensive reviews on chemometric approaches as well as available Raman instrumentation aimed at biological samples, the reader is directed to the listed references [30,31,37,41,42].

## 3. Secondary and Tertiary Structure

**Figure 3:**
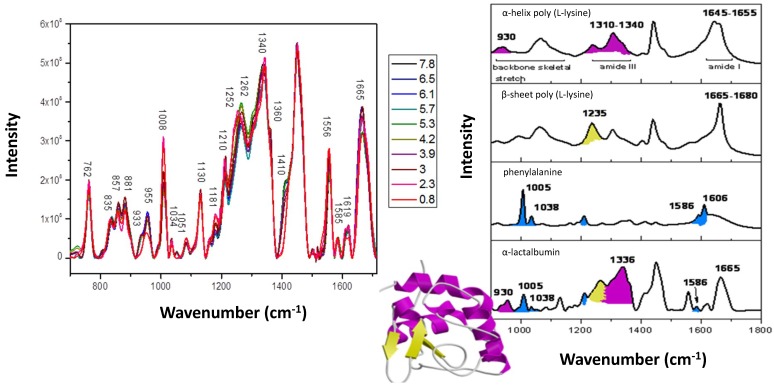
(**a**) pH-induced changes in the Raman spectra of lactalalbumin from pH 0.8–7.8, (**b**) Shaded peaks in the Raman spectra of selected polypeptides and phenylalanine, assigned to specific protein structure: -α-helix (purple), β-sheet (yellow) and aromatic residues (blue) [44].

A key cause of protein aggregation is the formation of non-native, partially unfolded intermediates with exposed regions capable of inter-molecular interactions [21,26,43]. The propensity of a protein to unfold, thereby exposing regions of residues, is not only influenced by changes in physiochemical conditions (e.g., temperature, pressure, pH and excipients) but can also be due to the intrinsic conformational properties of the protein (e.g., primary sequence, secondary and tertiary structure) [16,21,26]. Consequently, in order to understand and reduce antibody aggregation, it is necessary to evaluate even the most subtle of structural changes. Figure 3 highlights the key Raman spectral features associated with either α-helical or β-sheet structure, as well as an example of pH-induced loss of secondary and tertiary structure in α-lactalbumin as the native structure unfolds [44]. Subtle differences can be observed in the spectra of α-lactalbumin as the unfolding protein forms a series of intermediate species observed between the pH ranges 0.8–3.6, 3.6–4.6 and 4.6–7.8 [44].

As previously demonstrated in Figure 2, the Raman spectrum of a typical antibody has numerous features that can be easily assigned to both secondary and tertiary structure conformation. The Raman peaks observed at ~960, 1245 and 1674 cm^−1^ assigned to β-sheet indicate that this is the major secondary structure of the antibody, whilst features in the regions of ~500–550 cm^−1^ assigned to disulfide conformations, 800–860 cm^−1^ assigned to hydrogen bonding state of tyrosine residues and the peak at ~1550 cm^−1^ assigned to tryptophan are all markers of tertiary structure [17,24,43]. Raman spectroscopy is therefore an ideal tool for the characterisation of not only the native antibody but also the partially or fully-unfolded intermediates that lead to the formation of aggregates, including providing insight into the actual aggregation mechanisms [13,40,43].

## 4. Aggregation Mechanisms

The ratio of native to less-ordered or non-native intermediates can vary depending on the propensity of a protein to unfold and the specific aggregation mechanism, which in turn controls aggregate population size and concentrations [12,14,28]. Three reported aggregation mechanisms include nucleation dominated (ND) where new aggregates are formed directly from the monomeric proteins, chain polymerization (CP) where partially unfolded monomers add onto existing aggregates and associated polymerization (AP), which occurs when existing aggregates combine to form larger ones [28,45,46,47]. By using a combination of Raman spectroscopy, circular dichroism (CD) and dynamic light scattering (DLS), Barnett et al. [13] were able to demonstrate differences in the extent of structural changes of anti-streptavidin IgG1 when ND aggregation was induced compared to CP or AD. Significant Raman spectral changes during ND where observed in the amide I region, in the tryptophan assigned peak at ~1550 cm^−1^ and in the 510–540 cm^−1^ region. Raman peaks in this latter region have been extensively used to determine changes in S-S stretching of the disulfide bonds as peak position is directly related to the gauche/trans conformers. The Raman peaks at ~508, 520 and 540 cm^−1^ are associated with GGG, TGG and TGT, respectively [48,49,50]. Through the comparative use of DLS, Barnett’s study also indicated that aggregates formed by ND were relatively smaller than those formed via the CP and CP/AP mechanisms, indicating that the most significant structural changes are related to the change from monomer to dimer to trimer, rather than being observed as aggregates grow in size by combining aggregates [13].

The combination of Raman spectroscopy and DLS was also applied to the investigation of aggregation mechanisms in the model protein lysozyme, providing complementary information that determined the degree of influence of aggregation on the reversibility of protein unfolding and refolding [24]. By relating changes in protein size to shifts in position of peaks observed in the amide I region alongside changes in the tyrosine Fermi doublet at 850 and 830 cm^−1^, they were able to determine that during heating, at pH 4, changes in lysozyme tertiary structure occurred before significant changes in either secondary structure or size [24]. The ratio of the two tyrosine peaks observed at ~830 and 850 cm^−1^ (Intensity_850_/Intensity_830_) in a protein spectrum is another well-established Raman marker of hydrophobicity and side chain solvent exposure. A drop in the ratio of the tyrosine Fermi doublet indicates a decrease in exposure and increased involvement of tyrosine residues as strong hydrogen bond donors, whilst an increase in ratio indicates the opposite effect. [51,52]. A lack of significant temperature-induced changes in secondary structure of lysozyme at pH 4 was attributed to the formation of small aggregates as opposed to more substantial changes observed at pH 7 where large lysozyme aggregates were recorded [24].

## 5. Perturbation-Induced Aggregation

One of the key advantages of Raman spectroscopy is the ability to monitor perturbation-induced structural changes as shown in Table 1. This potentially provides insight into antibody stability of not only newly engineered antibodies but throughout the whole production process, right through to the formulated product.

Gómez et al. [40] demonstrated the sensitivity of Raman spectroscopy to differences in heat-induced conformational transitions in IgG4 variants. By comparing Raman spectral data sets of each heated IgG4 variant against the native stable antibody, it could be determined if unfolding only, aggregation only or both processes occurred. In particular, the Raman regions of ~760–770 and 875–880 cm^−1^ assigned to tryptophan, displayed the most significant changes indicating the extent of solvent exposure [53,54]. During heating, variations in Raman peak intensity indicated that tryptophan residues became initially less exposed as the F_ab_ domain of the antibody unfolded, followed by increased solvent exposure as the F_c_ domain unfolded; however, if aggregation occurred, there was further loss of exposure, suggesting changes in orientation of tryptophan residues within the F_c_ domain depending on the IgG4 thermal response [40].

Zhou et al. [22] also investigated temperature-induced spectral variation but their study investigated a formulated IVIG stored in a 250 nM glycine buffer. Although a key advantage of Raman spectroscopy is its sensitivity to structural and chemical composition, this also means that any recorded spectra will contain signals from all components of the sample; therefore, the spectrum of a protein in a buffer will contain signal from the protein and buffer, with buffer peaks potentially masking important protein peaks. In order to overcome this problem with the IVIG in glycine, the difference spectrum was determined by subtracting the spectrum acquired at 20 °C from the protein spectra acquired at different temperatures during heating [22]. From the difference spectra, they were able to resolve increased intensities in the β-sheet assigned peak at 1668 and 1686 cm^−1^ with heating as well as substantial peak shifts from the tyrosine Fermi doublet and tryptophan peak at ~1550 cm^−1^, again identifying specific changes in both secondary and tertiary structure during aggregation of the antibody [22]. This approach using the difference spectra was also successfully used by Webster et al. [55] to analyze Raman spectra acquired of insulin subjected to shear flow for 90 min. By subtracting the unsheared insulin spectrum from the sheared ones recorded, they were able to determine changes in marker peaks at 1678, 1630 and 1625 cm^−1^, indicating an increase in intermolecular H-bonded β-sheet structure of the protein [55]. Insulin aggregates were also investigated in formulated products using difference spectra, where spectral contributions of both glycerol and insulin were subtracted from the Raman spectrum of the heat stressed insulin [56]. The most significant spectral changes with heating were observed at 1675 cm^−1^ suggesting that this Raman peak is an important marker for protein aggregation.

## 6. Raman Optical Activity

**Figure 4:**
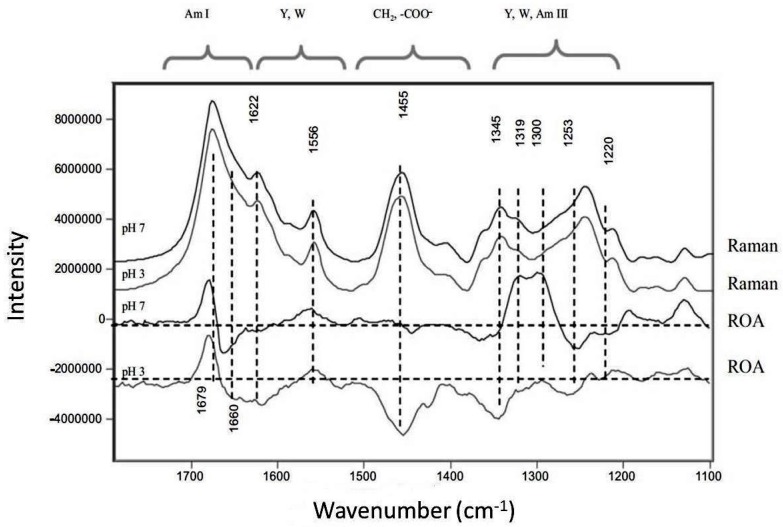
Raman and ROA spectra of IgG1 at pH 3 and 7 showing significant changes in the amide I and III regions for ROA spectra only. Reproduced with permission from [43]. Copyright Bentham Science Publishers Ltd. (2009).

Although Raman spectra has been extensively demonstrated as a sensitive tool for determining structural changes, some secondary and tertiary structural changes in proteins can be hard to determine in antibodies due to the dominance of the β-structure features in the spectrum, masking very subtle changes [22,25]. In a pH-induced study of IgG1 using FT-Raman spectroscopy, no variations in either the amide I or the amide III regions were observed, suggesting that secondary structure remained stable; however, when the alternative Raman technique of Raman optical activity (ROA) was applied, differences in these regions could be observed in the ROA spectra (Figure 4) [43]. ROA measures a small difference in intensity of Raman scattering from chiral molecules in right- and left- circularly polarized light and/or a small circularly polarized component in the Raman scattered light. It is therefore extremely sensitive to the most rigid parts of the protein [39,57]. It has been extensively used to investigate a range of biomolecules, including unfolding and aggregation in proteins [58,59,60,61,62,63,64]. In a pH-induced study of human IgG, variations observed in the ROA spectra of IgG1 and IgG2 at pH 7 and pH 3 revealed that whilst the secondary structure of the antibody remains relatively unchanged, significant changes in tertiary structure could be determined. Significant spectral differences were observed for the tyrosine and phenylalanine peak at 1622 cm^−1^, the tryptophan assigned peaks at 1556, 1455 and 1345 cm^−1^ and the tyrosine peak at 1220 cm^−1^, suggesting that the aromatic side chain environment changes at acidic pH with unfolding of the tertiary structure [43].

**Figure 5:**
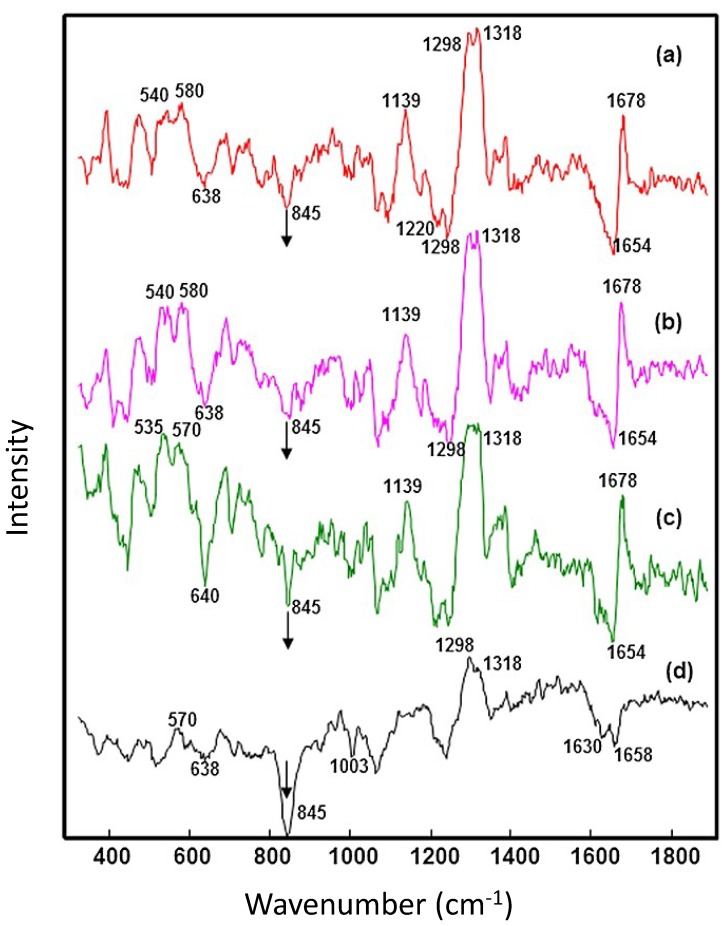
Background subtracted normalised ROA spectra of human monoclonal antibody IgG4(mAb1) (**a**) control (**b**) after 1-week thermal stress (**c**) after 2-week thermal stress (**d**) after 4-week thermal stress. Reproduced with permission from [65] Copyright © 2015 John Wiley & Sons, Ltd.

ROA has also been used to investigate temperature-induced aggregation in IgG4 in the presence of formulation excipients [65]. In the same way that buffers and excipients can mask Raman spectral features arising from the protein, ROA spectral features from the formulation placebo were also observed to dominate the ROA spectrum of the IgG4 [65]. As with the previously discussed Raman studies, the formulation placebo spectrum was subtracted from the spectra of the heated IgG4 revealing significant peaks in the amide I region assigned to β-sheet, the positive peaks at 1300 and 1320 cm^−1^ and negative peaks at 1200 and 1250 cm^−1^ (Figure 5). As the sample was heat-stressed over four weeks, there was a loss of intensity in the ROA peaks, indicating a loss of both secondary and tertiary structure [65]. 

## 7. Deep UV Resonance Raman

Another alternative Raman approach that has been used in the study of protein conformation is deep UV resonance Raman spectroscopy (DUVRR) spectroscopy. DUVRR spectroscopy has excellent sensitivity to structural changes in proteins and by tuning the laser within the UV region (180–260 nm), very specific information can be achieved. UV excitation also has the advantage of enhancing the Raman signal by a factor of 10^3^–10^5^ over conventional Raman scattering through the resonance effect [30]. In resonance spectroscopy, if the energy of the incident laser is within the molecular absorption peaks of chromophores or aromatics in the molecules, the signal can be enhanced, in the case of proteins resulting in a spectrum dominated by amide vibrations and aromatic amino acid peaks. One form of aggregation that has been extensively studied by DUVRR is the formation of fibrils [66,67,68,69,70,71]. By using a combination of 204 and 198 nm, Xiong et al. were able to assess changes in hydrogen bonding during polypeptide aggregation and fibril formation due to peak shifts observed in the amide I and amide II regions [70]. The excitation wavelength of 220 nm has also been used to determine two distinct stages of aggregation and fibril formation in the Tau protein by identifying variations in the DUVRR spectral features associated with an increase in β-sheet and loss of less-ordered structure, including a shift in peak position of the 1238 cm^−1^ to 1230 cm^−1^ [72]. In another study of fibrils, the sensitivity of the phenylalanine peak at ~1000 cm^−1^ to resonance enhancement was used to monitor spectral differences in the DUVRR spectra of hen egg white lysozyme (HEWL) fibrils compared to HEWL in its native conformation [67]. The fibril spectra showed a significant decrease in the peak intensity at ~1000 cm^−1^ compared to native lysozyme [67]. Further studies demonstrated that the loss of intensity observed with the fibrils was greater than the loss of intensity that was observed for the soluble phase of incubated lysozyme [66,68]. This distinction was linked to a greater exposure of the fibrils to water, highlighting the capacity of this Phe peak to probe solvent exposure as well as aid sample identification.

Although to date DUVRR spectroscopy has been under-utilized in the investigation of antibody aggregation, Bueno et al. investigated thermal unfolding of Rituximab using DUVRR spectroscopy at an excitation wavelength of 205 nm [73]. Rituximab is an IgG1 and was studied in a formulation containing polysorbate 80; however, unlike other Raman techniques, the resonance enhancement of the DUVRR spectra resulted in a spectrum dominated by the protein rather than the excipient. In particular, a peak shift, along with a broadening of the peak, was observed from ~1670 to 1660 cm^−1^ with heating indicating an increase in less-ordered structure [73].

## 8. Conclusions

As demonstrated, Raman spectroscopy is a powerful technique for the analysis of antibody aggregates, providing dynamic information about secondary structure, tertiary structure, and aggregation mechanisms. Raman features can clearly be used to identify changes, including solvent exposure of residues, conformation, molecular interaction and hydrogen bonding. The interpretation of these parameters can facilitate the elucidation of structural variations associated with aggregated antibodies or the aggregation process itself when measurements are made in-situ. This information can be readily obtained with minimal sample preparation from liquids samples, even at high concentrations as well as solid samples, thus making it feasible to directly compare and consistently monitor different states of an antibody sample.

The capacity of Raman spectroscopy for the structural analysis of antibodies and their aggregates throughout the development and formulation process, including end-use conditions, has the potential to generate valid predictions for candidate selection and optimization during drug development. In addition, during the manufacturing process, spectral markers of aggregation could be used to assure and monitor product quality.

A number of alternative Raman spectroscopic techniques, including ROA and DUVRR variant techniques, have intrinsic qualities that seek to overcome some of the challenges of conventional Raman spectroscopy in addition to providing tailored or specific information. A wider application and targeted development of Raman techniques can facilitate rigorous benchmarking with other established techniques and consequently their acceptance for routine use in the analysis of antibody aggregates.

## Figures and Tables

**Table 1:** Raman peaks highlighting perturbation-induced structural changes.

Peak (cm^−1^)	Spectral Change	Sample (Perturbation)	Structural Implication	Ref
**510/531/547** (S-S stretch)	Observable peaks at 510,531 & 545 in aggregate spectra vs. 531 for soluble Fc	Fc (unspecified)	GGG, TGG and TGT conformations present in aggregates vs mainly TGG in soluble Fc	[43]
	Increased perturbation in (I_510_/_540_) for ND aggregates	Anti-streptavidin IgG1(temperature)	ND aggregation pathway showed greater sensitivity to this marker.	[13]
**850/830** (Tyr)	No change	rhuMab * (Lyophilisation)	-	[25]
	Downshift	IVIG * (temperature)	Change in tertiary structure	[22]
	Increased ratio	Rabbit IgG (concentration)	Increased molecular interaction	[23]
**770/875** (Trp)	Increased/decreased intensity	IgG4 variant (temperature)	Less exposure to solvent/more exposure to solvent	[40]
**1000** (Phe)	Increased intensity	Rabbit IgG (storage)	Pre-aggregation	[23]
**1230** (β-sheet)	Broadening and shift to 1245 cm^−1^	Human IgG1 (pH)	Formation of intermolecular β-sheet	[43]
**I**_1340_/**I**_1360_ (Trp)	Decreased ratio	IVIG (temperature)	Reduced hydrophobicity (change in tertiary structure)	[22]
**1555** (Trp)	Downshift	IVIG/temperature	Change in tertiary structure	[22]
	Redshift	Anti-streptavidin IgG1(temperature)	Change in tertiary structure	[13]
**1630** (β-structure)	Increased/decreased intensity	IgG4 variant (temperature)	Increase in intermolecular H-bonding of β-structure/Loss of bonding or loss of secondary structure	[40]
**1669** (β-sheet)	Decreased intensity	Human IgG1(pH)	Loss of Intramolecular β-sheet	[43]
**1686** (β-sheet)	Increased intensity	Human IgG1(pH)	Intermolecular β-sheet formation	[43]

* Recombinant humanized monoclonal antibody (rhuMab), intravenous immunoglobulin (IVIG).
